# The effect of anchor attraction on the buying behavior of carbon labeled agricultural products: incorporating anchor attraction into TPB

**DOI:** 10.3389/fpsyg.2026.1948256

**Published:** 2026-09-11

**Authors:** Fei Wang, Nan Wang, Dehua Zhang

**Affiliations:** 1 School of Finance, Harbin University of Commerce, Harbin, China; 2 China School of Banking and Finance, University of International Business and Economics, Beijing, China

**Keywords:** anchor attraction, behavioral choice, carbon labeled agricultural products, environmental psychology, SEM

## Abstract

Carbon emissions from agriculture can be reduced by adding carbon labels on agricultural products. The growing trend of live streaming agricultural products is a significant tool for promoting low-carbon consumption. We investigates the influence of anchor attraction on consumer purchases of carbon-labeled agricultural products by including it in the theory of planned behavior. A total of 402 questionnaires were gathered via the Hangzhou resident questionnaire survey, and they were examined using a structural equation model. The findings show that attitude, subjective norms, perceived behavior control, and anchor attraction all have a direct impact on consumers’ intentions to buy carbon-labeled agricultural products. Purchase intention and perceived behavior control have a direct impact on purchase behavior. Purchase intention is significantly influenced by anchor attraction indirectly through attitude, perceived behavior control, and subjective norms. Product knowledge plays a moderating role in the relationship between purchase intention and actually purchase behavior, and low product knowledge can better promote the transformation from intention to behavior, which may be related to the current state of implementation of China’s carbon labeling system. This study develops a novel theoretical framework and, in a sense, makes up for the lack of attention paid to anchor attraction’s effect on consumers’ decisions. Our findings not only help mitigate carbon emissions from agricultural production, but also help guide low-carbon consumption and achieve carbon neutrality.

## 1 Introduction

In the process of production and daily life, humans emit excessive carbon dioxide, so that the concentration of carbon dioxide in the atmosphere continues to increase, resulting in global warming, polar glacier melting and climate change. In the face of serious global warming and climate anomalies, all countries are committed to transforming the mode of economic growth, advocating low-carbon economy and making efforts to reduce carbon emissions (Liu et al., 2023). The production process of agricultural products in China will emit a lot of carbon dioxide, among which, the greenhouse gas released during the production of food alone accounts for 26–34% of the global emissions of greenhouse gasses, and the growing demand for agricultural products will further increase the pressure on the environment (Crippa et al., 2021). Carbon labeling of agricultural products is an effective way to achieve more sustainable production and consumption.

Carbon labeling is a way to label a product with a quantitative indicator of greenhouse gasses emitted (Thogersen and Nielsen, 2016). Rather than being a law, carbon labeling is a policy or strategy to inspire producers and consumers to support and safeguard the environment. By understanding the carbon information on the product label, consumers are not only able to make informed judgments, but they can also take part in the effort to reduce emissions. China began to promote the “carbon footprint labeling” program in early 2018, and China’s carbon labeling system is also being developed and gradually improved. Considering that people’s knowledge of carbon-labeled agricultural products is limited and China’s carbon labeling program is still in its early stages, live streaming has become an important means to promote carbon labeled agricultural products into the public’s vision.

Live streaming e-commerce is a new business model that makes use of live streaming technologies on internet platforms to offer immersive product displays and information services to assit and guide shoppers (Lin et al., 2018). More and more people like to do live shopping through e-commerce (Chen et al., 2021). In China, 515 million people use e-commerce regularly, making up 48.2% of all Internet users. Anchors are the main group who dominate online live broadcast marketing activities (Li et al., 2024). Anchors encourage consumers to buy goods by displaying products and interacting with them, and their goal is to attract more viewers and maximize consumers’ buying behavior (Mao et al., 2022). Therefore, anchor attraction is an important feature that plays a decisive role. Anchor attraction can influence consumer psychological states and shape consumer cognition in the live streaming e-commerce environment (Zhu L. et al., 2021). It can also affect the opinions, decisions, and actions of most consumers (Zhao Y. Y. et al., 2018), thus becoming a key factor affecting consumer purchase decisions (Guo et al., 2022). Researching how anchor attraction affects consumers’ decisions to purchase carbon-labeled agricultural products would be interesting.

By combining past studies, we found that in terms of label types, the majority of research to date focused on nutrition and safety-related labels of food, while few focused on carbon-labeled products (Xu et al., 2024). As for content, prior study has mostly examined consumers’ attitudes toward carbon label products and if they are prepared to pay more for them (Chen et al., 2024; Gadema and Oglethorpe, 2011; Yang and Lin, 2024). Previous studies disregarded how anchors influenced consumers’ decisions. In the study on the buying behavior of carbon label products (Laksmawati et al., 2024; Guo et al., 2022), the attraction of anchors, an important influencing factor, was ignored. From the perspective of theoretical basis, the UTAUT and the theory of innovation diffusion are applied to the study of the acceptance and adoption behavior of carbon label products (Liu et al., 2023; Wong et al., 2020). To fill gaps in the above studies, this paper introduces anchor attraction into TPB to explore the impact of anchor attraction on the buying behavior of carbon labeled agricultural products in the Chinese context.

In this paper, anchor attraction is introduced into TPB to examine how anchor attraction influences the purchase behavior. Using product knowledge as a moderating variable, the broader factors affecting the purchasing behavior of carbon labeled agricultural products were studied. Thus, a new theoretical framework is constructed, which can provide new ideas for the subsequent research. Our research helps to cultivate citizens’ awareness of low-carbon lifestyles, achieve “everyone’s carbon reduction, everyone’s benefits”, and also provides insights for the promotion of carbon-labeled agricultural products, providing new marketing strategies for live streaming platforms. Moreover, the results of this research contribute to elucidating the influence of product information disclosure on the evolution of production practices.

This paper has the following three innovative points: (1) Anchor attraction is included in TPB, supplementing the previous literature that ignored the impact of anchor attraction on the purchasing behavior of carbon labeled agricultural products. (2) The direct and indirect impacts of anchor attraction on purchasing behavior were explored, as well as the mediating role of psychological factors. (3) By investigating the moderating influence of product knowledge, the broader determinants influencing purchase behavior were examined.

The structure of this paper is as follows: The second chapter includes a literature review. The third chapter contains the paper’s theoretical framework and hypotheses proposed. The research methodologies are in the fourth chapter. Chapter Five presents the results analysis and hypothesis testing, along with the verification of data authenticity. Chapter Six provides a discussion of the results. The conclusion is covered in the seventh chapter.

## 2 Literature review

### 2.1 Research on carbon labeled agricultural products

(1) China’s carbon labeling system started late, and only a portion of agricultural products are currently labeled with carbon. The public’s awareness of carbon labeling only increased following China’s proposal of the “3,060” double carbon objective and its gradual emergence in enterprise consciousness. However, since China implemented a carbon labeling system in 2018, carbon labeling has been developed and widely spread in China due to the increasing environmental awareness of producers and consumers. Chinese carbon labeling system primarily for goods and services related to electronics now (Xu and Lin, 2022). In China, farming products such as fruits, vegetables, crops, and dairy products have not yet fully embraced carbon labeling (Xu et al., 2024).
(2) China’s carbon label certification technology continues to develop. When the carbon labeling system was first established in China, it was mostly established and implemented using foreign techniques (Li et al., 2017). Although China’s carbon labeling system has not been fully popularized, it has already involved electronic and electrical products and a variety of agricultural products (Xu and Lin, 2022). In 2018, the Carbon Footprint Quantification Requirements and Guidelines for Greenhouse Gas Products were released, providing an effective supply for increasing carbon-related certification systems. In 2021, the designation and launch of the Implementation Rules for the Voluntary Evaluation of Product Carbon Labels for Unified Implementation of the Industry marked a major breakthrough in the evaluation and certification of China’s carbon labels, and the entire industry can carry out the evaluation and certification of carbon labels in accordance with the rules. The certification system for carbon labels is also being improved. At present, the products of many enterprises have been certified carbon labels and obtained certificates.
(3) Carbon labeling is essential to achieve sustainable production and consumption. Carbon labeling is one of the most effective solutions for reducing CO2 emissions from agricultural production (Chen et al., 2024). Carbon labeling reflects the level of CO2 emissions associated with the production process, which is designed to encourage producers to support and protect the environment (Li et al., 2017). It gives producers the chance to carry out an extensive evaluation of the environmental effect of their services or goods over their whole life cycle, which can encourage them to produce more responsibly (Zhao et al., 2017). Carbon labeling provides a tool that allows consumers to understand the carbon footprint, thus encouraging them to buy low-carbon products (Roa-Goyes and Pickering, 2024). Through carbon labeling, consumers can understand sustainability information, make the source of carbon emissions more apparent, and influence changes in public consumption patterns (Wu et al., 2014). One useful strategy for promoting low-carbon development is carbon labeling, which has emerged as a realistic method for society to take action against climate change (Zhao R. et al., 2018). Carbon labeling is therefore essential to achieve more sustainable production and consumption.

### 2.2 Research on anchor attraction

Anchor attraction refers to the ability of an anchor to attract viewers, which is based on multiple dimensions, including physical appearance, personal traits, and social interaction (Li et al., 2024). A charismatic anchor can capture audience attention, with the goal of attracting more viewers and maximizing consumer purchasing behavior (Mao et al., 2022). When anchor attraction is strong, it enhances user interest in and attention to the relevant brands and products (Yin and Wang, 2022). Li et al. (2024) showed that anchor attraction can stimulate positive emotions in consumers, which in turn further drive consumer impulse to purchase. Yin and Wang (2022) proposed that anchors who display products from multiple angles and levels can help consumers dispel doubts. Such authentic viewing experiences, directly obtained by consumers, generate a greater sense of genuine trust than recommendations from any other channel (Liu et al., 2025). Research by Liu et al. (2025) also indicates that anchor attraction can evoke positive emotions and trust among consumers. The personal charisma of anchors also helps facilitate the establishment of social relationships with consumers and influences consumer attitudes and purchase behavior (Lin et al., 2018).

Anchor attraction can influence consumer psychological states and shape consumer cognition in the live streaming e-commerce environment (Zhu P. et al., 2021). It can also affect the opinions, decisions, and actions of most consumers (Zhao Y. Y. et al., 2018), thus becoming a key factor affecting consumer purchase decisions (Guo et al., 2022). Anchors play a crucial role in stimulating consumer purchase desires (Zhu L. et al., 2021; Zhu P. et al., 2021) and can make significant contributions to product promotion by retailers (Zhang, 2023). Existing studies have extensively explored the facilitating effects of anchor characteristics, including attractiveness, on consumer purchase intentions and behavior. For example, in the agricultural products sector, well known anchors often rely on their fan bases and credibility to promote agricultural sales, enhancing consumer impulse buying tendencies during the live streaming process (Yu et al., 2025). Shi et al. (2026) found that anchor characteristics significantly influence consumer impulse buying behavior for agricultural products in live streaming contexts. Wang et al. (2025) explored the factors influencing live stream shoppers’ purchase decisions for tourism and hospitality products. The findings indicate that service convenience significantly impacts impulse buying through perceived value and e-trust. Currently, e-commerce live streaming is in a phase of rapid development, thus requiring substantial theoretical and empirical data to assess its impact. The key role of anchor attraction has yet to be explored in research.

### 2.3 Literature review

Our review of the literature shows that prior research has two main shortcomings. (1) The important factor of anchor attraction is ignored in the relevant studies. Carbon-labeled agricultural products have not been well promoted, and live broadcast platforms are an important means to promote carbon-labeled agricultural products into the public vision. Attractive anchors can attract attention and maximize the incentive for consumers to buy carbon-labeled agricultural products. (2) There have been extensive studies on the characteristics of anchors, but no separate studies have been conducted on the impact of anchor attraction and purchase behavior of carbon-labeled agricultural products. Therefore, this paper incorporates anchor attraction as a key factor to investigate its impact on the purchasing behavior of carbon-labeled agricultural products.

## 3 Theoretical framework and hypothesis proposed

### 3.1 Theoretical basis

#### 3.1.1 Theory of Planned Behavior

From the perspective of the Theory of Planned Behavior (TPB), whether people engage in a specific action is influenced by both motivational and non-motivational factors. The motive factor is people’s behavioral intention. The stronger intention to carry out an action, the higher the possibility that the action will actually be carried out. In contrast to the Theory of Rational Behavior (TRA), behavior also depends, at least in part, on non-motivational factors (Ajzen, 1991). Based on TRA, Ajzen introduced the variable of perceived behavior control.

TPB has strong explanatory power in predicting sustainable development behavior, and has been widely used in many fields (Lou et al., 2022; Wang et al., 2023). Many researchers have applied TPB to the study of consumers’ purchasing behavior (Liu et al., 2020; Chaudhary and Bisai, 2018), including the purchasing behavior of carbon-labeled products (Liu et al., 2022).

#### 3.1.2 Extended theory of planned behavior

TPB had strong explanatory and predictive power, as evidenced by the researchers’ demonstration that its three anthems could account for 27% of behavior variance and 39% of intention variance. However, a large number of variances that the conventional TPB model was unable to explain (Conner and Armitage, 1998). The researchers extended TPB to improve its explanatory and predictive power. Extended TPB is frequently used in a number of environmental protection domains, such as green agricultural production (Li et al., 2023), green consumption (Wang et al., 2024), waste classification (Lou et al., 2022), green electricity (Harorli and Ercis, 2023), low-carbon travel (Zhang et al., 2023), low-carbon products (Taufique and Vaithianathan, 2018). TPB has become one of the most widely used theoretical models in the field of environmental protection, so it is considered a suitable theoretical basis for this study.

**Figure 1:**
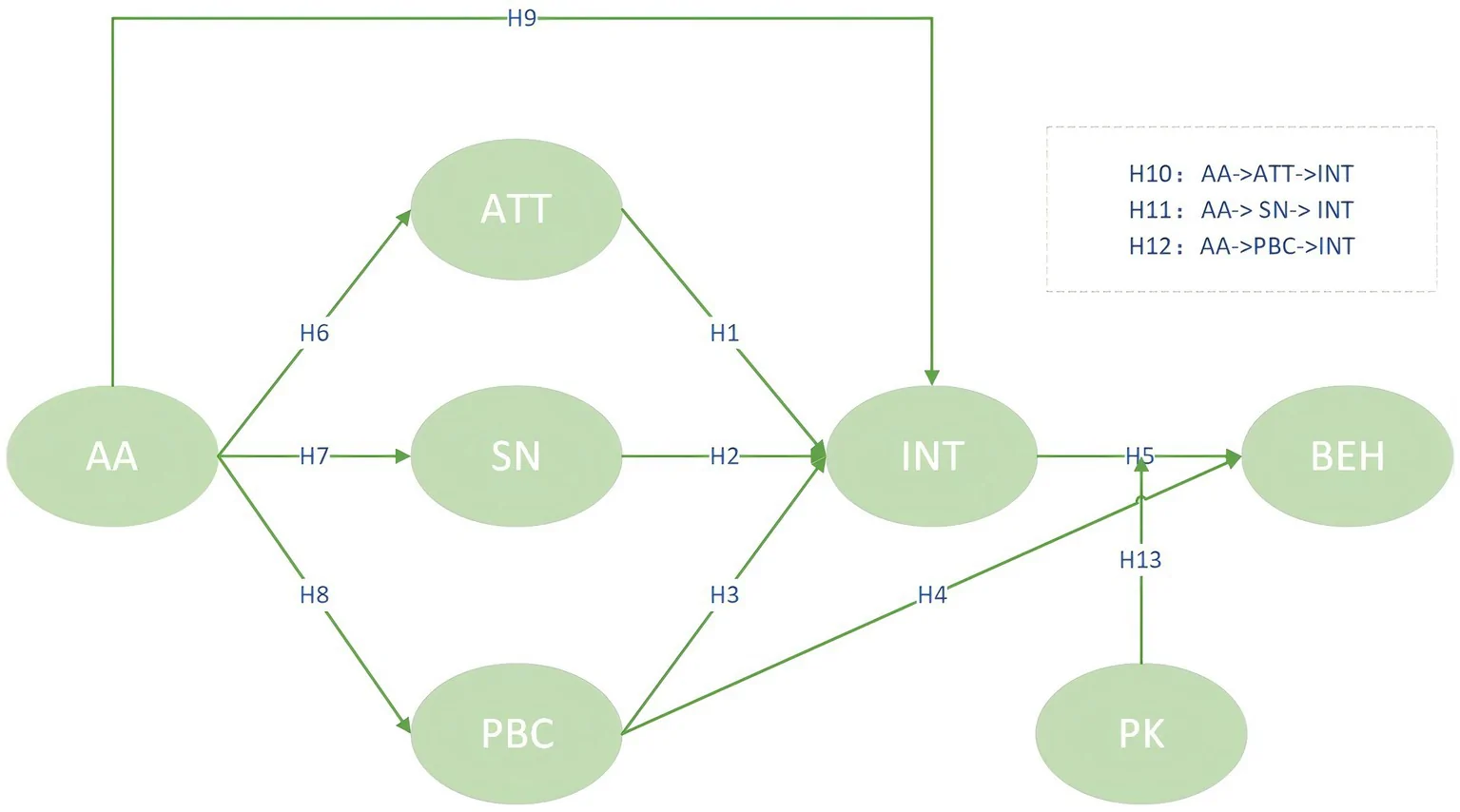
Conceptual model.

This paper introduces anchor attraction into TPB as a prevariable to explore its influence on the purchasing behavior of carbon-labeled agricultural products. Anchor characteristics are the key factors affecting consumers’ purchasing decisions, and anchor attraction is one of the important anchor characteristics. Studies have shown that the professional knowledge and personal appeal of anchors can affect consumers’ attitudes and purchasing behaviors. People tend to think positively about people who are socially attractive. An attractive host can attract the attention of more viewers and maximize the stimulation of buying behavior. The specific conceptual model diagram is shown in Figure 1.

### 3.2 Hypothesis proposal

#### 3.2.1 Attitude

Attitude (ATT) is defined as a tendency exhibited by some degree of approval or disapproval of a particular entity. It is generally understood as a state of mind that produces favorable or unfavorable evaluations of a particular behavior. Many studies confirm that attitude is one of the most powerful predictors of environmental behavior (Taufique and Vaithianathan, 2018). Consumer purchase intentions are significantly influenced by attitudes, with a positive attitude probably translating into a more proactive buying intention. Researchers generally discovered a positive correlation between attitude and green purchase intention (Wang et al., 2024; Nekmahmud et al., 2022).

The attitude in this paper is defined as the degree of approval or disapproval evaluation of the behavior of purchasing carbon-labeled agricultural products on live streaming platforms. Based on our research, individuals who hold the belief that purchasing products with a carbon label has a favorable effect on the climate and environment will be more inclined to support this. So we hypothesize:

*H1*: Attitude has a positive impact on the purchase intention.

#### 3.2.2 Subjective norms

Ajzen (1991) defines subjective norms (SN) as a factor that is related to social pressure that is associated with the implementation or non-implementation of a behavior. If a person has strong subjective norms, they may change their decisions because of the support or lack of support from others. On the contrary, if a person is not sensitive to pressure from significant others, then the opinions of others will not affect their decision making. In the study of green purchasing behavior, researchers generally discovered a favorable association between subjective norms and purchase intention (Nekmahmud et al., 2022; Xu et al., 2020). Some studies have also proved that subjective norms has positive effects on low-carbon behavior (Wang et al., 2022).

The subjective norms in this paper refer to the opinions of people in the social environment of the respondents on the purchase behavior of carbon-labeled agricultural products on the live streaming platform. Based on our research, if people around and significant others support the behavior of purchasing carbon-labeled agricultural products, or they often buy carbon-labeled agricultural products on live streaming platforms, they will play a role model and support the respondents’ views. On the contrary, if the people around them do not support the behavior, have a bad evaluation of the behavior, or the people around them do not do it, it will have the opposite effect on the respondents. So we hypothesize:

*H2*: Subjective norms has a positive impact on the purchase intention.

#### 3.2.3 Perceived behavior control

Perceived behavior control (PBC) is defined as how easy or difficult it is to assess the specific behavior based on past experience and expected difficulties and obstacles. In the study of green purchasing behavior, researchers generally found a favorable association between perceived behavior control, purchase intention and behavior (Sun et al., 2022; Liang et al., 2024). Consumers’ past experience is a good predictive factor of their behavioral intention, and researchers have found that perceived behavior control positively affects low-carbon behavior in food selection (Li et al., 2017).

PBC refers to the perceived ease and expected barriers to purchasing carbon-labeled agricultural products on the live streaming platform in this article. If people think that buying carbon-labeled product on live streaming platforms is difficult and out of their control, then people will not act in this way. On the contrary, individuals are more inclined to behave in this way if they think the behavior is easy, within their control, and easily integrated into everyday life. So we hypothesize:

*H3*: Perceived behavior control has a positive impact on purchase intention.

*H4*: Perceived behavior control has a positive impact on purchase behavior.

#### 3.2.4 Purchase intention

Behavioral intention refers to an individual’s judgment of the subjective probability of taking a certain behavior. The stronger the intention of a person, that is, the more inclined he is to carry out a certain behavior. Customers’ willingness to buy ecologically friendly products is a common indicator of their green purchase behavior, and eventually influences their decision to buy such products in order to support environmental sustainability (Han et al., 2022). In the study of green purchasing behavior, researchers generally found a favorable association between purchase intention and behavior (Sun et al., 2022; Yadav and Pathak, 2017).

Purchase intention (INT) in this article refers to the behavioral tendency to buy carbon-labeled agricultural products. Based on our research, individuals who are inclined to purchase agricultural products with a carbon label are more likely to do so. Therefore, we make the hypothesis:

*H5*: Purchase intention has a positive effect on purchase behavior.

#### 3.2.5 Anchor attraction

The anchor attraction (AA) refers to its ability to attract viewers, and this is based on a number of aspects, including their looks, personal qualities, and social interaction. An attractive anchor can attract more audience’s attention (Li et al., 2024). Anchor attraction is one of the most important anchor characteristics and a key factor affecting consumers’ purchasing decisions (Li et al., 2024). Anchor attraction can stimulate consumers’ purchasing behavior to the maximum extent (Zhu L. et al., 2021). The anchor’s personal traits can influence consumers’ psychological state and shape their perceptions as well as their purchasing decisions within the live-streaming e-commerce environment (Zhu P. et al., 2021).

When anchor attraction is strong, it enhances users’ interest in and attention to related brands and products (Yin and Wang, 2022). Zhu L. et al. (2021) indicates that an anchor’s personal charisma influences consumers’ attitudes and behaviors. During live streaming, anchors can generate high-quality dialogues with consumers through product demonstrations and shopping guidance (Akdevelioglu and Kara, 2020). By explaining the health and environmental benefits of carbon-labeled agricultural products, anchors enable consumers to perceive the purchase of such products as beneficial, thereby fostering positive attitudes.

Subjective norm refers to the perceived social pressure when individuals perform a certain behavior. During live streaming, viewers actively participate through comments, likes, gift giving, and purchases, which provides them with a real time interactive experience. Zhang et al. (2023) indicates that a highly interactive relationship between the anchor and consumers can alter consumers’ cognition and emotions, deepen their familiarity with both the products and the anchor, and enhance trust. With a foundation of trust, a stronger tendency toward normative conformity is more likely to emerge. Moreover, during the viewing process, consumers also perceive pressure from behavioral expectations arising from the anchor and the fan community, which may therefore exert a positive influence on subjective norms.

Perceived behavior control refers to an individual’s subjective perception of the ease or difficulty of performing a specific behavior. In the context of live streaming e-commerce, anchors with stronger attraction tend to possess more sophisticated live streaming rhetoric and product explanation skills, enabling them to clearly convey the certification information, advantages, features, and purchasing channels of carbon-labeled agricultural products. This reduces the time cost for consumers to gather information, makes them perceive that purchasing such products is easy, and thus may positively influence perceived behavioral control.

Through solid theoretical research, some researchers have found that anchor attraction has an impact on consumers’ behavioral intentions (Han and Xu, 2020). Anchors with exquisite appearance can impress the audience deeply, thus attracting more loyal fans to watch their live streaming. Previous studies have proved that anchors with better appearance have higher physical attraction, which can generate the audience’s trust in them, and stimulate the audience’s purchase intention through the intermediary effect of trust (Yu et al., 2025). Since our study included anchor attraction into TPB for the first time, in order to fully explore the mechanism of anchor attraction’s influence on buying intention and behavior, we hypothesized:

*H6*: Anchor attraction has a positive influence on attitude.

*H7*: Anchor attraction has a positive impact on subjective norms.

*H8*: Anchor attraction has a positive influence on perceived behavior control.

*H9*: Anchor attraction has a positive impact on purchase intention.

*H10*: Anchor attraction has a positive indirect effect on purchase intention through attitude.

*H11*: Anchor attraction has a positive indirect effect on purchase intention through subjective norms.

*H12*: Anchor attraction has a positive indirect effect on purchase intention through perceived behavior control.

#### 3.2.6 Product knowledge

Product knowledge (PK) in this paper measures the degree of consumer knowledge about carbon- labeled agricultural products. Research has shown that few customers have knowledge of carbon labels and how to use them to contribute to the environment (Hartikainen et al., 2014). Customers’ acceptance of and comprehension of carbon labeling affect their inclination to purchase low-carbon products. Currently, even if consumers are aware of low-carbon policies, it can be challenging for them to comprehend the many indications on carbon labels, which could have an impact on their choice of products. To encourage green consumption, knowledge can help consumers recognize and select green consumption activities (Lee et al., 2006). In order to close consumers’ intention-behavior gap, consumers’ vulnerabilities need to be taken into account (Nieto-García et al., 2024). Considering the late establishment of China’s carbon labeling system and the lack of relevant knowledge of carbon-labeled agricultural products, this paper takes product knowledge as a moderating variable to explore how it can promote the transformation of consumers’ purchase intention to behavior. Therefore, we propose the following research question:

*H13*: How does product knowledge moderate the relationship between purchase intention and purchase behavior?

The specific assumptions are proposed as shown in Figure 1.

## 4 Methodology

### 4.1 Research methods

We used SPSS Statistics 26 for descriptive analysis. To investigate the potential correlation, a two-stage approach of SEM is used. AMOS 24.0 is used to assess model fit and hypothesis testing. Compared with the traditional multiple regression model, SEM has many advantages. First, SEM can simultaneously examine the correlational relationships between multiple independent variables and multiple dependent variables. Secondly, SEM can solve the endogeneity problem (Cao and Yang, 2017). Thirdly, SEM also considers mediation analysis, we tested the moderating impact of product knowledge using the SPSS PROCESS macro.

### 4.2 Questionnaire design

The TPB measurement scale was locally revised and improved based on the findings of prior research, along with the pertinent policies and the state of the carbon label system’s implementation for residents in Zhejiang Province. Since the previous research had almost nothing to do with it, we contacted two sustainability experts to assess the study questionnaire. We adjusted the survey questions in response to their feedback. Vague or unfamiliar terms are avoided to reduce the difficulty of sample groups. After a small range of pretests, the final questionnaire was determined. The survey has been divided into eight sections, with a total of 34 questions. The second to eighth parts were measured by Likert seven-point scale, as shown in Table 1.

**Table 1:** Questionnaire.

Constructs	Items
Anchor attraction (AA)	The anchor’s appealing appearance or voice enhances my affection for the anchor (Li et al., 2024).
	I find the anchor’s personality to be admirable (Li et al., 2024).
	The unique live streaming style of the anchor aroused my interest in shopping (Li et al., 2024).
	The sincere and kind attitude of the anchor makes me want to watch the live streaming of the anchor (Li et al., 2024).
	I think that communicating with this anchor is simple (Zhu L. et al., 2021).
Attitude (ATT)	Buying carbon-labeled agricultural products is a good idea (Nautiyal and Lal, 2022).
	Buying carbon-labeled agricultural products is enjoyable (Nautiyal and Lal, 2022).
	Purchasing agricultural products with carbon labels is beneficial to the environment (Lou et al., 2022).
	Buying carbon-labeled agricultural products can be very rewarding (Wang et al., 2024).
Subjective norms (SN)	My family recommended me to buy carbon-labeled agricultural products (Lou et al., 2022).
	My friend advised me to buy carbon-labeled agricultural products (Lou et al., 2022).
	A media campaign would push me to buy carbon-labeled agricultural products (Lou et al., 2022).
	Individuals whose viewpoints I respect are more likely to advocate for me to buy carbon-labeled agricultural products (Nautiyal and Lal, 2022).
Perceived behavior control (PBC)	Buying carbon-labeled agricultural products seems easy to me.
	I do not think it’s difficult for me to purchase carbon-labeled agricultural products.
	I have the knowledge to buy carbon-labeled agricultural products.
	I can afford to buy carbon-labeled agricultural products.
Product knowledge (PK)	I have a good understanding of carbon-labeled agricultural products compared to regular products (Eberle et al., 2022).
	I can tell if a certain product has a carbon label on it (Eberle et al., 2022).
	I understand the policy of carbon-labeled agricultural products.
	I know the benefits of carbon-labeled agricultural products (Eberle et al., 2022).
	I know more about carbon-labeled agricultural products than the people around me.
Purchase intention (INT)	I plan to purchase agricultural products with a carbon label (Sun et al., 2023).
	I intend to pay more for carbon-labeled agricultural products (Liu et al., 2023).
	I have very strong intentions to buy carbon-labeled agricultural products within the next 2 weeks (Nekmahmud et al., 2022).
Purchase behavior (BEH)	I often buy carbon-labeled agricultural products.
	I always purchase carbon-labeled agricultural products.
	I would purchase carbon-labeled agricultural products compared to other products of the same type (Dorce et al., 2021).

### 4.3 Research location and sample

#### 4.3.1 Research location

**Figure 2:**
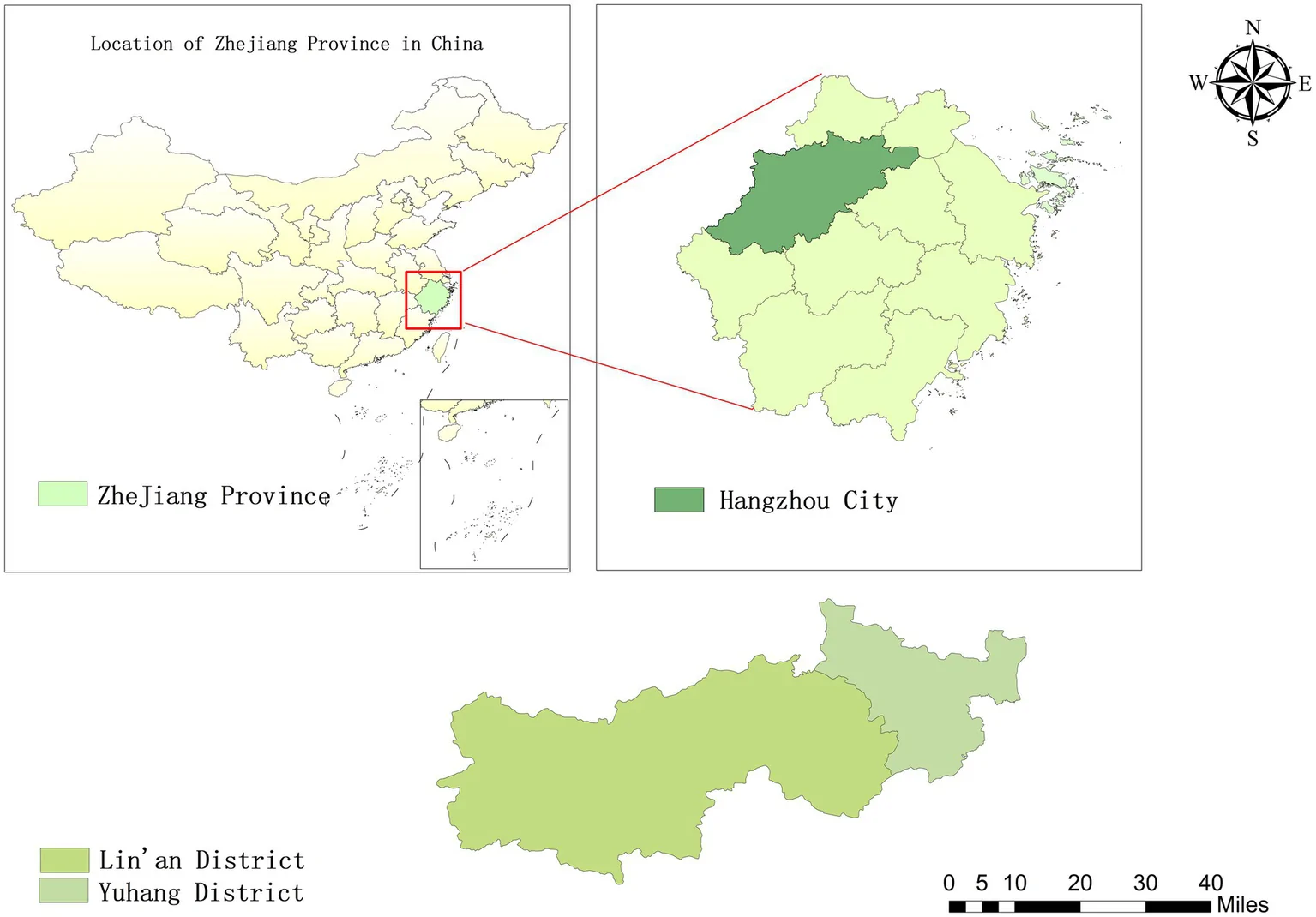
Study area.

Hangzhou, referred to as “Hang,” is the capital city, vice-provincial city and megacity of Zhejiang Province, located in East China. The reasons for selecting Hangzhou as the research site in this paper are as follows. First, policy advantages. 2024-06-28 Notice of Hangzhou Municipal People’s Government on issuing the Implementation Plan of the National Carbon Peak Pilot Project (Hangzhou). Its contents include that gradually expand the coverage of carbon labels, and orderly promote the promotion and application of carbon labels and carbon labels in the field of consumption. This policy provide a solid institutional guarantee for the market development of carbon-labeled agricultural products. Second, a mature consumer market. Hangzhou has a developed economy, high resident income levels, and progressive consumption concepts, with strong acceptance and willingness to purchase green and low-carbon products. The city has numerous consumers with favorable economic conditions and advanced environmental awareness. Third, leading practice in carbon labeling. Hangzhou is an important birthplace of carbon-labeled agricultural products in China. Fourth, representative sample areas. This study selects residents from Yuhang District and Lin’an District of Hangzhou as the survey sample. The first carbon label for agricultural products in China was created in Lin’an City. Lin’an City continues to explore and develop in the field of carbon labeled agricultural products, there are a variety of agricultural products have been labeled with carbon labels. Yuhang District is the core area of Hangzhou, GDP ranks first, with a good prospect of carbon labeled agricultural products sales. The characteristics of the research area are shown in Figure 2.

#### 4.3.2 Data and samples

Two Hangzhou districts’ residents were chosen as samples for this study. Our survey used a combination of online and offline methods to collect data. For online surveys, a questionnaire is provided through the online survey tool. Conduct surveys of users who have purchased carbon-labeled agricultural products on live streaming platforms, and questionnaires using anonymous survey links that can be shared. To ensure that respondents have relevant experience in evaluating anchor attraction, the first question of the questionnaire is a screening item: “Have you ever purchased carbon-labeled agricultural products on a live streaming platform?” Only consumers who have actually placed orders for such products during live streams are retained to proceed to the formal questionnaire. For offline investigations, face-to-face interviews are used, and field investigations are conducted in supermarkets, stores and other places that sell carbon-labeled agricultural products. We believe that this group may be interested in carbon-labeled agricultural products, and we will also use the above screening question to filter consumers who have actually placed orders for such products during live streams. The data from online surveys can be imported directly into SPSS for descriptive analysis, and the data from offline surveys is manually entered into SPSS by team members and assigned to other members for inspection to ensure accuracy. The survey was conducted from March 10, 2024 to April 25, 2024, and a total of 500 questionnaires were distributed, with 402 valid questionnaires. A valid questionnaire excludes that take too short a time to answer, that choose the same option in succession, and that have important information missing. The complete questionnaire is presented in the supplementary materials.

To mitigate the potential threat of common method bias (CMB), firstly, we said at the beginning of the questionnaire that each person just needs to respond according to their particular circumstances, and the survey is anonymous. Second, we guarantee to respondents that the information we get will only be utilized for our scientific studies and will not be shared with outside parties. Third, determine whether the questionnaire has to be explained or dictated based on the respondents’ age and educational background. Fourthly, this survey set a time limit of 10–15 min to avoid prevent interviewers from providing fictitious or nonsensical responses. These measures were implemented in line with recommended practices for controlling CMB (Podsakoff et al., 2003; Podsakoff et al., 2003).

Furthermore, this study employed Harman’s single-factor test to determine whether a single factor could account for the majority (over 50%) of the variance (MacKenzie and Podsakoff, 2012; Podsakoff et al., 2003). Among the seven factors extracted based on eigenvalues greater than 1, the maximum variance explained by a single factor was 45.917%; therefore, it can be reasonably concluded that common method variance did not affect the research findings (Podsakoff et al., 2003).

## 5 Research results

### 5.1 Descriptive analysis

The fundamental traits of the Hangzhou inhabitants under investigation in this study are displayed in Table 2. 52.74% were male and 47.26% were female, indicating a balanced ratio between men and women. The age distribution is basically balanced. The majority of respondents (79.59%) had completed high school or further, and the interviewees’ high educational attainment is consistent with China’s adoption of a nine-year mandatory education program. As for monthly income, respondents <9,215 yuan accounted for 34.83%, respondents 9,215–20,442 yuan accounted for 26.12%, respondents 20,442–32,195 yuan accounted for 17.91%, and respondents over 32,195 yuan accounted for 21.15%, indicating that the income level of Hangzhou residents is relatively high. The specific situation is shown in Table 2.

**Table 2:** Sample demographics.

Measure	Item	Percentage (%)
Gender	Male	52.74%
	Female	47.26%
Age	18–34	22.39%
	35–49	25.62%
	50–64	24.88%
	65 and above	27.11%
Level of education	Never went to school	6.72%
	Primary school	8.71%
	Middle school	4.98%
	High school	1.99%
	Junior college	36.57%
	Undergraduate college	33.33%
	Postgraduate	7.70%
Income (yuan/month)	Under 9,215	34.83%
	9,215–20,442	26.12%
	20,442–32,195	17.91%
	Above 32,195	21.14%
Family population	1	11.19%
	2	22.14%
	3	25.87%
	4	18.91%
	5	9.95%
	6 and above	11.94%

### 5.2 Reliability and validity analysis

#### 5.2.1 Reliability analysis

Before evaluating the structural model, we first evaluate the measurement model. The results of confirmatory factor analysis (CFA) were as follows: CMIN/DF = 1.601, GFI = 0.932, AGFI = 0.913, RMSEA = 0.039, NFI = 0.947, RFI = 0.938, IFI = 0.980, TLI = 0.976, CFI = 0.979. SPSS26 was used to analyze the reliability of each variable (Table 3). Every construct has a Cronbach’s Alpha coefficient larger than 0.7, it shows that the reliability meets the standard (Kiliç, 2016). The CR value is also an indicator of reliability. The CR values of all the constructs were within the recommended range, from 0.810 to 0.916 (Bagozzi and Yi, 1988) in the study.

**Table 3:** Cronbach’s alpha.

Constants	Measurement item	Cronbach’s alpha
Anchor attraction	AA1	0.919
	AA2	
	AA3	
	AA4	
	AA5	
Attitude	ATT1	0.902
	ATT2	
	ATT3	
	ATT4	
Subjective norms	SN1	0.892
	SN2	
	SN3	
	SN4	
Perceived behavior control	PBC1	0.879
	PBC2	
	PBC3	
	PBC4	
Product knowledge	PK1	0.902
	PK2	
	PK3	
	PK4	
	PK5	
Purchase intention	INT1	0.823
	INT2	
	INT3	
Purchase behavior	BEH1	0.836
	BEH2	
	BEH3	

#### 5.2.2 Validity

This study has undergone three types of validity tests: discriminant validity, convergence validity, and content validity. Content validity is an important embodiment of the quality of the scale. In this study, expert adjustment and empirical methods were used to revise the scale according to the expert group’s review opinions on the survey draft and questions. Convergence validity was tested for the homogeneity of the dimensions by mean variance extraction (AVE). AVE value should be greater than 0.5 (Fornell and Larcker, 1981); all AVE values met the threshold (Table 4). The square root of AVE of each dimension is greater than the absolute value of the relationship between the dimensions, which indicates good discriminant validity (Fornell and Larcker, 1981) (Table 5).

**Table 4:** Convergence validity.

Constructs	Measurement item	Unstd.	Std.	CR	AVE
Anchor attraction (AA)	AA1	1	0.835	0.916	0.687
	AA2	1.002	0.834		
	AA3	0.968	0.841		
	AA4	0.948	0.817		
	AA5	0.952	0.816		
Attitude (ATT)	ATT1	1	0.844	0.902	0.698
	ATT2	0.981	0.843		
	ATT3	0.932	0.805		
	ATT4	0.999	0.848		
Subjective norms (SN)	SN1	1	0.866	0.894	0.678
	SN2	0.937	0.842		
	SN3	0.8	0.762		
	SN4	0.949	0.819		
Perceived behavior control (PBC)	PBC1	1	0.714	0.887	0.665
	PBC2	1.056	0.749		
	PBC3	1.32	0.902		
	PBC4	1.28	0.879		
Intention (INT)	INT1	1	0.774	0.810	0.587
	INT2	1.036	0.763		
	INT3	0.948	0.761		
Behavior (BEH)	BEH1	1	0.875	0.839	0.638
	BEH2	0.947	0.833		
	BEH3	0.714	0.674		
Product knowledge (PK)	PK1	1	0.829	0.902	0.648
	PK2	1.071	0.884		
	PK3	0.993	0.790		
	PK4	0.973	0.767		
	PK5	0.943	0.749		

**Table 5:** Discriminant validity test.

	PK	BEH	INT	PBC	SN	ATT	AA
PK	**0.805**						
BEH	0.596	**0.799**					
INT	0.576	0.676	**0.766**				
PBC	0.541	0.609	0.591	**0.815**			
SN	0.625	0.639	0.641	0.603	**0.823**		
ATT	0.587	0.618	0.586	0.573	0.642	**0.835**	
AA	0.616	0.597	0.588	0.551	0.634	0.626	**0.829**

Note: The bolded content represents the square root of AVE for each dimension.

### 5.3 Model fit test

Amos26 was used to perform the fit test prior to the path analysis. Compared with χ2, χ2/df is relatively robust. CMIN/DF is less than 5, the model and data fits well. When GFI and AGFI are greater than 0.9, the model fits well, and greater than 0.8 belongs to the acceptable range (Abdullah et al., 2014). RMSEA should in the range of 0.05–0.08 (Browne and Cudeck, 1992). The analysis showed that the data were well model-fitted as all indicators met the recommended thresholds (Table 6).

**Table 6:** Model fit.

Fit index	Value
CMIN/df	2.157
RMSEA	0.054
GFI	0.906
AGFI	0.883
NFI	0.927
TLI	0.953
IFI	0.959

### 5.4 Path analysis result

**Figure 3:**
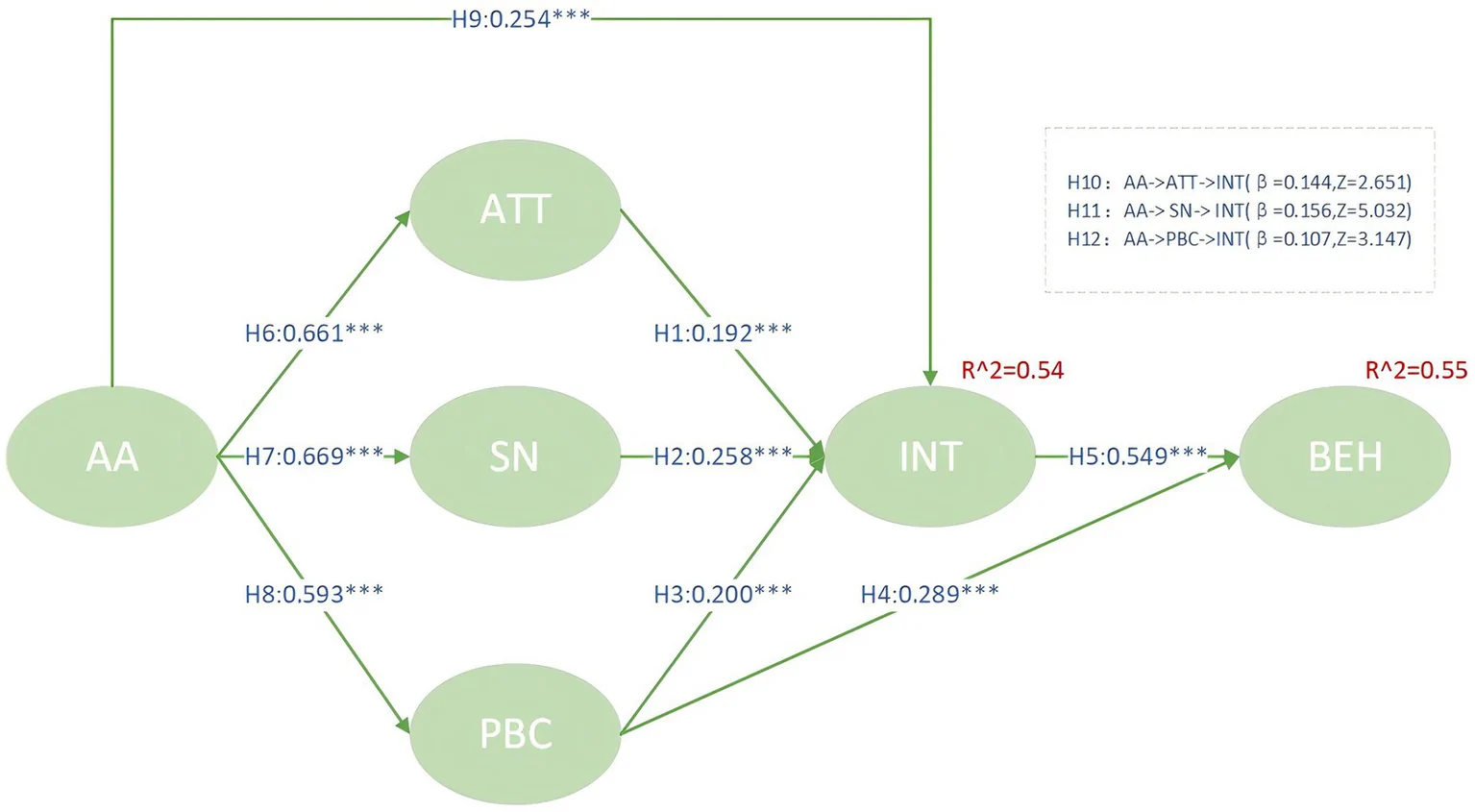
Standardized output of constructs. Note: ***Significant at *p* < 0.001 level.

The results of the hypothesis test are show in Figure 3. The results show that the standardized regression coefficients of all direct effects are significant, supporting hypotheses H1-H9.

In addition to examining the direct effects, we also examine the indirect effects, having verified hypotheses H10-H12. Set the specific syntax in Amos to get the product of the coefficients, and percentile bootstrapping and bias-corrected percentile bootstrapping 1,000 times at the 95% level. Since there are three paths in this paper, we first test whether the total indirect effect of anchor attraction on purchase intention is significant (Table 7). As shown in Table 8, the results of the Bootstrap test support the hypotheses H10, H11, and H12 by confirming that anchor attraction indirectly influences consumers’ intentions to purchase carbon-labeled agricultural products through ATT, SN, and PBC.

**Table 7:** Total indirect effect.

Estimate	Bias-corrected		*p*	Estimate	Percentile		*p*
	Lower	Upper			Lower	Upper	
0.378	0.300	0.481	0.001	0.287	0.287	0.468	0.002

**Table 8:** Mediator effect test results.

Parameter	Estimate	SE	*Z*	Bias-corrected		Percentile	
				Lower	Upper	Lower	Upper
AAtoATTtoINT	0.114	0.043	2.651	0.034	0.205	0.030	0.200
AAtoSNtoINT	0.156	0.031	5.032	0.105	0.236	0.099	0.224
AAtoPBCtoINT	0.107	0.034	3.147	0.039	0.176	0.040	0.176

**Figure 4:**
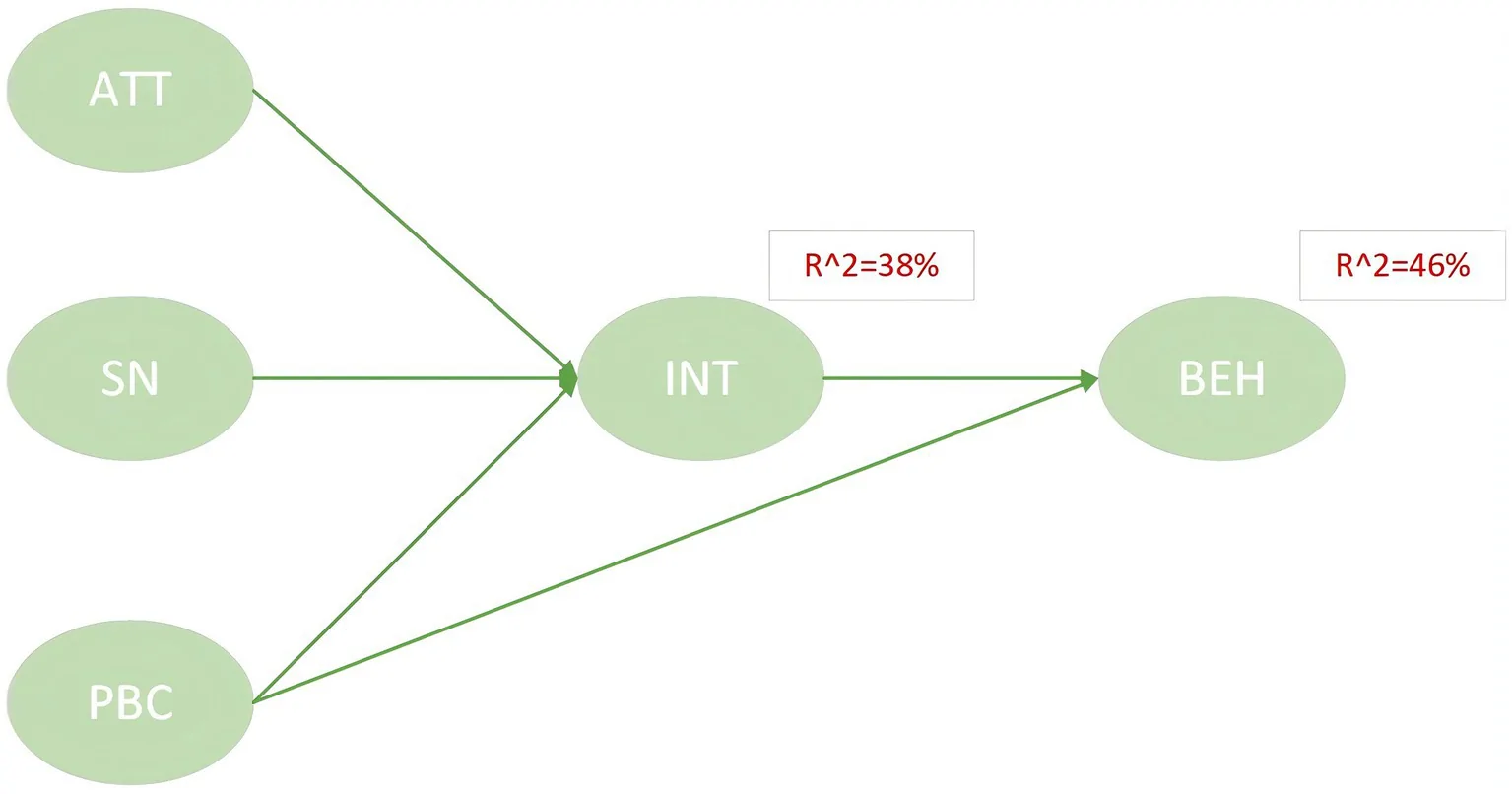
Explanatory power of the original TPB.

Using AMOS26 software, we compared the explanatory power of the original TPB model with the TPB model extended with anchor attraction (Figure 4). Original TPB model explain 38% of the behavior intention variance and 46% of the behavior variance, while the TPB model established in this study explains 54% of the behavior intention variance and 55% of the behavior variance, and the explanatory ability of the model is significantly improved. In the remaining literature that employs the Extended TPB framework for research, the model’s predictive accuracy for behavior and behavioral intention typically ranges between 30–40% (López-Mosquera et al., 2014; Yadav and Pathak, 2017). Furthermore, the CMIN/df value for the original TPB model was 4.194, whereas for the improved model it was 2.157, indicating a significant improvement in fit. This finding suggests that the TPB model created in the present study is superior to the original TPB model for evaluating consumer behavior related to carbon-labeled agricultural products.

### 5.5 Moderate test results

**Figure 5:**
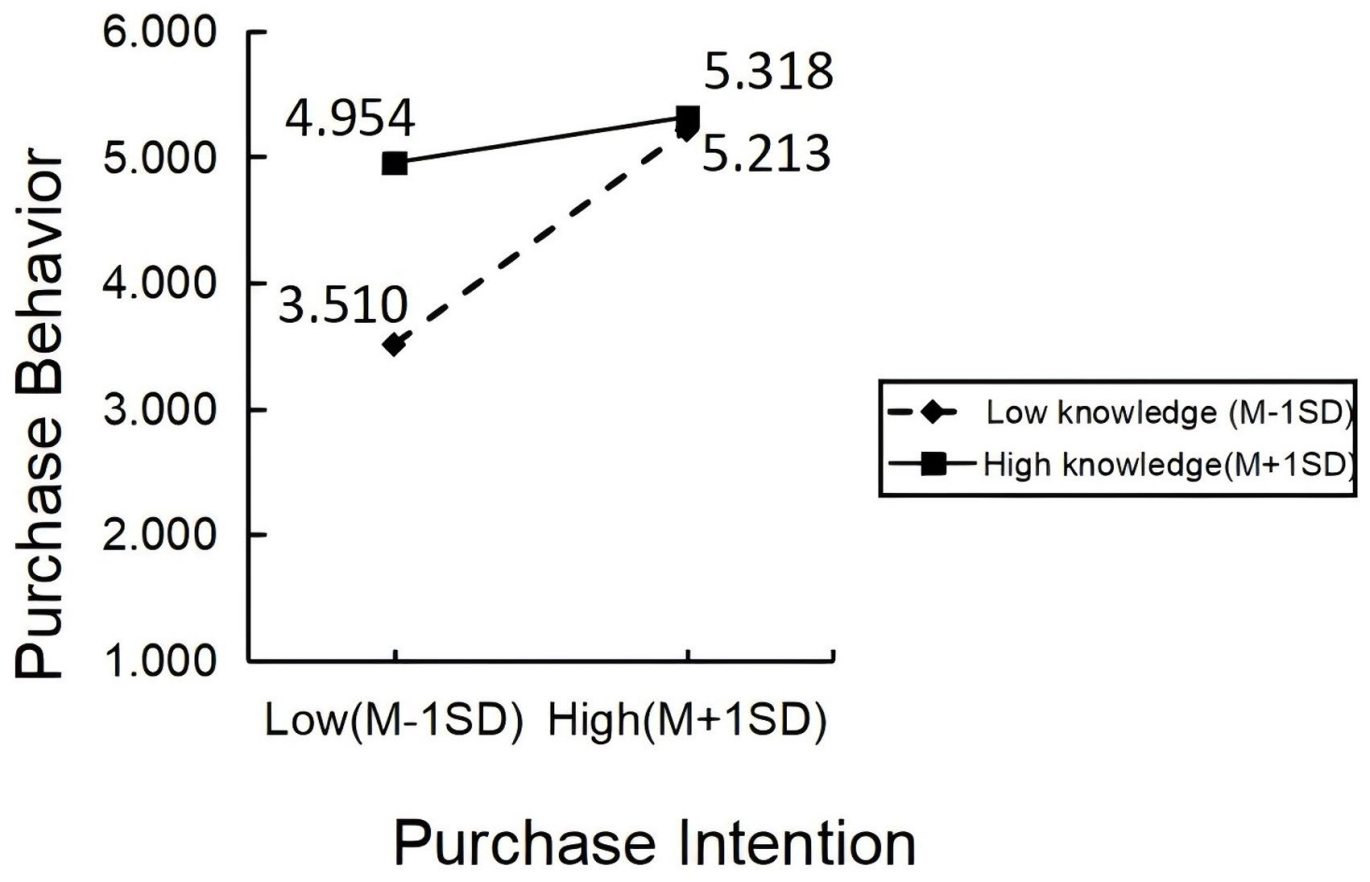
Simple slope diagram.

For the moderating test, we used Model1 in the PROCESS macro to examine the moderating effect of product knowledge. In order to generate interaction terms, this study conducted 5,000 bootstraping under 95% confidence interval (Preacher and Hayes, 2008). The three components of PK, INT, and BEH were first given standardized values. The interaction variable values were then computed and confirmed, and AMOS v26 was used to validate the structural model. We found that product knowledge plays a moderating role in the relationship between intention and behavior (*β* = −0.171, *p* < 0.05) (Table 9). A simple slope diagram of the moderating test is shown in Figure 5.

**Table 9:** Moderation analysis results.

	Coeff	SE	T	*p*	LICI	UICI
Constant	4.749	0.061	77.435	0.000	4.628	4.869
INT	0.361	0.045	7.932	0.000	0.271	0.450
PK	0.284	0.048	5.880	0.000	0.189	0.378
Int_1	−0.171	0.028	−6.144	0.000	−0.226	−0.116

Specifically, this study examined the effect of purchase intention on purchase behavior at high (M + 1SD) and low (M − 1SD) levels of product knowledge. As shown in Figure 5, the slope of the line for low product knowledge (*β* = 0.594, *p* < 0.001) is larger than that for high product knowledge (*β* = 0.128, *p* < 0.001), indicating that higher product knowledge unexpectedly weakens the translation of purchase intention into actual behavior. In other words, compared with the low product knowledge condition, the positive effect of purchase intention on purchase behavior is significantly attenuated when product knowledge is high, suggesting that product knowledge negatively moderates the transformation from purchase intention to purchase behavior.

## 6 Discussion

(1) ATT, SN and PBC had significant positive effects on purchase intention (*β* = 0.192, *p* < 0.05; *β* = 0.258, *p* < 0.05; *β* = 0.200, *p* < 0.05). Hypothesis H1, H2, and H3 are supported. Consistent with previous studies, ATT, SN and PBC are important determinants of purchase intention (Li et al., 2017; Xu et al., 2024). This indicates that positive evaluations of the behavior of purchasing carbon-labeled products, and peer influence, knowledge, and past experience can increase people’s positive purchase intentions. Education is the most effective tool to improve residents’ awareness of environmental protection. When implementing the carbon labeling system, China should provide scientific and accessible education about carbon labeling, so as to improve residents’ awareness and understanding and generate positive evaluation on low-carbon consumption. Strengthening social publicity and cultural construction, creating a social atmosphere of low-carbon consumption is also beneficial to improve residents’ awareness of carbon reduction, and promote green consumption and purchase. Making it easier to buy carbon-labeled agricultural products could also reduce the perceived barriers to low-carbon consumption.
(2) Both perceived behavior control and purchase intention had significant positive effects on purchase behavior (*β* = 0.289, *p* < 0.05; *β* = 0.549, *p* < 0.05), supporting hypothesis H4 and H5. Consistent with previous research, past experience and anticipated barriers are key factors in purchase behavior (Mabkhot, 2024), and consumers with a strong intention to purchase low-carbon products are more likely to generate actual purchase behavior (Gunarathne et al., 2020). This suggests that consumers’ relevant knowledge, perceived convenience of purchase, and their purchase intention are key determinants that translate into actual purchasing behavior. It is suggested that the sales side should further explore and expand the sales channels of carbon-labeled agricultural products. This is beneficial to reduce consumers’ perceptions of difficulties in purchasing carbon-labeled agricultural products. The use of live streaming channels to narrow the distance with young consumers, the use of newspapers, television and other media to strengthen the elderly’s awareness of carbon-labeled agricultural products.
(3) Anchor attraction had significant positive effects on ATT, SN and PBC (*β* = 0.661, *p* < 0.05; *β* = 0.669, *p* < 0.05; *β* = 0.593, *p* < 0.05), supports hypothesis H6, H7, and H8. Purchase intention was very positively impacted by anchor attraction, which supports hypothesis H9 (*β* = 0.254, *p* < 0.05). Consistent with previous studies, features such as the attractiveness of anchors can affect consumers’ psychology and shape consumers’ perceptions and purchasing decisions in the live streaming e-commerce environment (Zhu P. et al., 2021). In addition, our research also found that anchor attraction had a significant positive impact on ATT, SN and PBC. Attractive anchors can enable consumers to have a positive evaluation of carbon-labeled products, generate peer effects, and reduce consumers’ perceived barriers. It is suggested that anchors enhance the interaction with the audience, establish interpersonal relationships like friends with consumers, and pay attention to dressing and grooming, improve their attractiveness and influence in the hearts of the audience, attract more viewers to watch live streaming and participate in live streaming, in order to promote sales.
(4) Anchor attraction has indirect influence on purchase intention through ATT, SN, and PBC (*β* = 0.114, *Z* = 2.651; *β* = 0.156, *Z* = 5.032; *β* = 0.107, *Z* = 3.147), support hypothesis H10-H12. Previous studies have reached the same conclusion. They believe that the characteristics of e-commerce anchors will influence consumers’ purchase intention, and consumers’ psychological state plays an intermediary role in this process (Han and Xu, 2020). This paper confirms once again that anchor attraction, an important anchor feature, has an indirect influence on the purchase intention through the psychological factors of consumers. When people are faced with market choices, they will be influenced by those who are in the same position around them. Our research creatively found that anchor attraction can promote the formation of social relationships with consumers, exert peer effects among consumers, and further promote purchase intention. In live streaming, interaction also takes place among viewers (Han and Xu, 2020), which may be another reason why the attractiveness of anchors can improve consumers’ social norms. In addition, our research also creatively found that anchor attraction can improve consumers’ perceived behavior control, and through professional explanation and vivid and intuitive product display, consumers’ perceived difficulty in purchasing carbon-labeled agricultural products can be reduced, thus improving consumers’ purchase intention. It is suggested that anchors enhance their personal attractiveness by improving talent display, personal narrative, interesting comments and other means, interact with consumers, pay attention to consumers’ psychological state when watching live streaming, improve the interest of live broadcast, and thus stimulate consumers’ purchase intention to the maximum extent.
(5) Product knowledge moderated the relationship between purchase intention and purchase behavior (*β* = −0.171, *p* < 0.05). Thus, we conclude H13, that is, product knowledge negatively moderates the facilitating effect of purchase intention on purchase behavior. Different from the study of previous (Shehawy, 2023), our study found that with the improvement of knowledge level, the influence of purchase intention on purchase behavior weakens, which is different from the conventional situation. It is possible that acquired information does not directly translate into concrete behaviors because different cultures and social norm (Rendon-Marin et al., 2024). China’s carbon labeling system was established relatively late. For most people, carbon labeling is a new term, and people’s general cognition of carbon labeling is low. In this context, a higher level of product knowledge may instead lead consumers to adopt a more cautious attitude toward carbon-labeled products, thereby weakening the translation of purchase intention into actual behavior. Another contributing factor is that the promotion and application of China’s carbon labeling system were not initially extended to the food industry (Xu and Lin, 2022). During the past few years, customers’ willingness to pay for products with a carbon label has drastically decreased. Further analysis shows that the change of WTP is mainly attributed to the change of consumer economic environment (Yang and Lin, 2024), which is one of the possible reasons for the weak effect of product knowledge. Therefore, this negative moderating effect is not a research bias, but rather a localized phenomenon resulting from the combined influence of the developmental stage of China’s carbon labeling system, consumers’ cognitive characteristics, and the macroeconomic environment. It is suggested that the focus of the next stage of carbon labeling policy work should be appropriately shifted to raising consumer awareness, advocating carbon reduction for everyone. Consumers should give priority to agricultural products with carbon labels when purchasing. We expect carbon-labeled agricultural products to be priced reasonably and carbon labeling policies to be better implemented without increasing costs.

## 7 Conclusion

Through empirical test, this study draws the following conclusions: First, The willingness to purchase agricultural products with a carbon label is significantly positively influenced by their ATT, SN and PBC. Second, purchasing behavior is influenced by both PBC and purchase intention. Thirdly, after introducing anchor attraction as a former variable, we found that anchor attraction has a significant positive impact on residents’ ATT, SN and PBC. Fourth, anchor attraction indirectly influences purchase intention through ATT, SN and PBC. Fifth, product knowledge has a moderating effect in the relationship between purchase intention and purchase behavior.

Theoretically, first, this study discusses consumers’ purchasing behavior of carbon-labeled agricultural products from the standpoint of anchor attraction. Previous studies have largely overlooked the significant role of live streaming platforms and anchor attraction in consumers’ purchasing decisions. Given that carbon-labeled agricultural products remain a novel concept for most consumers, live streaming has emerged as an effective medium for raising public awareness of these products. Meanwhile, as the central figure and key opinion leader in live streaming commerce, an attractive anchor can capture viewers’ attention and facilitate purchasing behavior. Second, this paper incorporates anchor attraction into TPB, creating a new theoretical framework. This study not only investigates the impact of psychological factors on consumers’ purchase intention and behavior toward carbon-labeled agricultural products, but, more importantly, identifies the positive facilitating effect of anchor attraction. This not only broadens the research direction for future studies, but also contributes to enriching the literature on carbon labeling. Third, examining the moderating role of product knowledge on purchase intention and purchase behavior can help identify the broader range of factors that influence purchasing behavior.

In fact: (1) From the perspective of anchor attraction and residents’ psychological factors, this research contributes to the advancement of China’s carbon label system, and works toward achieving carbon neutrality. This study identifies the critical role of residents’ psychological factors in shaping their purchase decisions regarding carbon-labeled agricultural products, which holds significant implications for the popularization and promotion of such products. In practice, first, the abstract value of low-carbon environmental protection should be transformed into concrete benefits that consumers can perceive, for instance, by clearly stating the amount of carbon dioxide emissions reduced through purchasing the product during live streaming, thereby enhancing consumers’ environmental self-efficacy and fostering a positive attitude toward carbon-labeled agricultural products. Second, it is recommended that live streaming platforms establish a certification mechanism for “low-carbon anchor” to encourage leading influencers to proactively recommend carbon-labeled agricultural products during their broadcasts, leveraging their fan base to stimulate consumers’ conformity buying behavior. Finally, it is advisable to set up dedicated zones for carbon-labeled agricultural products on major e-commerce platforms, adopting intuitive graphical labels to replace complex carbon footprint data, so as to reduce consumers’ perceived difficulty in understanding. (2) Starting from the live streaming platform, this paper explores the impact of anchor attraction, an important anchor characteristics, on the purchasing behavior of labeled agricultural products, which helps to expand the sales and dissemination channels of carbon-labeled agricultural products. The study identifies the critical role of streamer attractiveness. During live streaming, anchors should fully utilize their personal appeal and the advantages of real time interaction on e-commerce platforms. They can attract viewers’ attention by enhancing their approachable image, incorporating prize winning Q&A sessions on carbon-label knowledge into the broadcast, and visiting the cultivation bases of carbon-labeled agricultural products. (3) This study identifies the negative moderating effect of product knowledge on the relationship between purchase intention and purchase behavior, which can be attributed to the relatively late introduction of China’s carbon labeling system and the possibility that high knowledge consumers may hold higher and more cautious selection criteria. It is recommended to strengthen the improvement and further promotion of the carbon labeling system, enhance the publicity and popularization of carbon labeling knowledge, lower consumers’ cognitive barriers, gradually cultivate consumers’ habits of purchasing carbon labeled agricultural products, and promote the development of low carbon agriculture and the formation of a low carbon consumption market for agricultural products. In this process, the focus should be on establishing a third party endorsement mechanism for carbon labeling certification and providing verifiable carbon emission reduction data, so as to alleviate the concerns of high knowledge consumers. (4) Consumers’ low carbon choices will be transmitted to the production side through market signals, driving producers to actively adjust their production methods and shift to agricultural production models with low carbon emissions. The findings of this study not only help increase the sales volume of agricultural products bearing carbon labels but also contribute to reducing carbon emissions at the production stage.

Although this paper provides valuable conclusions, there are still some limitations: (1) Because of the limited amount of influencing elements this article examines, it’s difficult to accurately represent the factors that influence purchasing behavior. In the future, more relevant theories will be used for in-depth verification, Such as the Value-Belief-Norm Theory, the Stimulus-Organism-Response Theory. (2) Hangzhou is the target of this study, which reflects a clear regional concentration in its sampling source. As a city with a highly developed economy and a vibrant digital economy, Hangzhou’s residents may possess higher income levels, greater consumption capacity, and stronger low-carbon awareness than the national average; this, to some extent, limits the external validity and generalizability of the study’s findings. Future research could expand the survey scope to include different regions and cities at various administrative levels across China for comparative analysis. (3) This study covers all age groups, and it is still necessary to conduct comparative studies on the purchasing behaviors of Generation X, millennials, and baby boomers when purchasing carbon-labeled agricultural products in the future. (4) Although it has been empirically verified that there is no significant common method bias, low-carbon consumption is a behavior with high social desirability, and respondents may tend to provide socially desirable answers. In addition, when filling out the questionnaire, respondents may keep their answers consistent due to the psychological motivation to maintain consistency, thereby still posing a risk of common method bias.

## Data Availability

The raw data supporting the conclusions of this article will be made available by the authors, without undue reservation.
