# Supplementary material for: The effect of anchor attraction on the buying behavior of carbon labeled agricultural products: incorporating anchor attraction into TPB
Source: Front Psychol. 2026 Sep 11;17:1948256. doi: 10.3389/fpsyg.2026.1948256 (PMC13612271; doi:10.3389/fpsyg.2026.1948256)
Supplement: Supplementary file 1 [file Supplementary_file_1.docx]

**Survey Questionnaire: The Impact of Anchor Attraction on Purchase Behavior of Carbon-Labeled Agricultural Products**

**Dear Respondent：**

This survey aims to investigate the influence mechanism of anchor attraction on consumers' purchase behavior of carbon-labeled agricultural products. The results of this survey will be used solely for academic research purposes. All responses will be collected anonymously. Please answer according to your actual situation. Thank you for your participation!

**Q0：Have you ever purchased carbon-labeled agricultural products on a live streaming platform?**

Yes (continue to answer) ☐	No (stop answering) ☐

**Part 1: Basic Information**

| Gender | ☐ Male ☐ Female |
| --- | --- |
| Age | ☐ 18-34 ☐ 35-49 ☐ 50-64 ☐ 65 and above |
| Level of education | ☐ Never went to school ☐ Primary school ☐ Middle school  ☐ High school ☐ Junior college ☐ Undergraduate college  ☐ Postgraduate |
| Income (yuan/month) | ☐ Under 9215 ☐ 9215-20442 ☐ 20442-32195 ☐ Above 32195 |
| Family population | ☐ 1 ☐ 2 ☐ 3 ☐ 4 ☐ 5 ☐ 6 and above |

**Part 2: Please indicate your opinions on the following statements based on your true feelings. Please note that all items are single‑choice questions. Please tick (√) in the corresponding box for each row, using the scale below: 1 = Strongly Disagree, 2 = Disagree, 3 = Somewhat Disagree, 4 = Neutral, 5 = Somewhat Agree, 6 = Agree, 7 = Strongly Agree.**

| **Items** | **1** | **2** | **3** | **4** | **5** | **6** | **7** |
| --- | --- | --- | --- | --- | --- | --- | --- |
| The anchor's appealing appearance or voice enhances my affection for the anchor. | ☐ | ☐ | ☐ | ☐ | ☐ | ☐ | ☐ |
| I find the anchor's personality to be admirable. | ☐ | ☐ | ☐ | ☐ | ☐ | ☐ | ☐ |
| The unique live streaming style of the anchor aroused my interest in shopping. | ☐ | ☐ | ☐ | ☐ | ☐ | ☐ | ☐ |
| The sincere and kind attitude of the anchor makes me want to watch the live streaming of the anchor. | ☐ | ☐ | ☐ | ☐ | ☐ | ☐ | ☐ |
| I think that communicating with this anchor is simple. | ☐ | ☐ | ☐ | ☐ | ☐ | ☐ | ☐ |
| Buying carbon-labeled agricultural products is a good idea. | ☐ | ☐ | ☐ | ☐ | ☐ | ☐ | ☐ |
| Buying carbon-labeled agricultural products is enjoyable. | ☐ | ☐ | ☐ | ☐ | ☐ | ☐ | ☐ |
| Purchasing agricultural products with carbon labels is beneficial to the environment. | ☐ | ☐ | ☐ | ☐ | ☐ | ☐ | ☐ |
| Buying carbon-labeled agricultural products can be very rewarding. | ☐ | ☐ | ☐ | ☐ | ☐ | ☐ | ☐ |
| My family recommended me to buy carbon-labeled agricultural products. | ☐ | ☐ | ☐ | ☐ | ☐ | ☐ | ☐ |
| My friend advised me to buy carbon-labeled agricultural products. | ☐ | ☐ | ☐ | ☐ | ☐ | ☐ | ☐ |
| A media campaign would push me to buy carbon-labeled agricultural products. | ☐ | ☐ | ☐ | ☐ | ☐ | ☐ | ☐ |
| Individuals whose viewpoints I respect are more likely to advocate for me to buy carbon-labeled agricultural products. | ☐ | ☐ | ☐ | ☐ | ☐ | ☐ | ☐ |
| Buying carbon-labeled agricultural products seems easy to me. | ☐ | ☐ | ☐ | ☐ | ☐ | ☐ | ☐ |
| I don't think it's difficult for me to purchase carbon-labeled agricultural products. | ☐ | ☐ | ☐ | ☐ | ☐ | ☐ | ☐ |
| I have the knowledge to buy carbon-labeled agricultural products. | ☐ | ☐ | ☐ | ☐ | ☐ | ☐ | ☐ |
| I can afford to buy carbon-labeled agricultural products. | ☐ | ☐ | ☐ | ☐ | ☐ | ☐ | ☐ |
| I have a good understanding of carbon-labeled agricultural products compared to regular products. | ☐ | ☐ | ☐ | ☐ | ☐ | ☐ | ☐ |
| I can tell if a certain product has a carbon label on it. | ☐ | ☐ | ☐ | ☐ | ☐ | ☐ | ☐ |
| I understand the policy of carbon-labeled agricultural products. | ☐ | ☐ | ☐ | ☐ | ☐ | ☐ | ☐ |
| I know the benefits of carbon-labeled agricultural products. | ☐ | ☐ | ☐ | ☐ | ☐ | ☐ | ☐ |
| I know more about carbon-labeled agricultural products than the people around me. | ☐ | ☐ | ☐ | ☐ | ☐ | ☐ | ☐ |
| I plan to purchase agricultural products with a carbon label. | ☐ | ☐ | ☐ | ☐ | ☐ | ☐ | ☐ |
| I intend to pay more for carbon-labeled agricultural products. | ☐ | ☐ | ☐ | ☐ | ☐ | ☐ | ☐ |
| I have very strong intentions to buy carbon-labeled agricultural products within the next two weeks. | ☐ | ☐ | ☐ | ☐ | ☐ | ☐ | ☐ |
| I often buy carbon-labeled agricultural products. | ☐ | ☐ | ☐ | ☐ | ☐ | ☐ | ☐ |
| I always purchase carbon-labeled agricultural products. | ☐ | ☐ | ☐ | ☐ | ☐ | ☐ | ☐ |
| I would purchase carbon-labeled agricultural products compared to other products of the same type. | ☐ | ☐ | ☐ | ☐ | ☐ | ☐ | ☐ |
